# It’s inequality, stupid!

**DOI:** 10.1007/s11609-021-00433-x

**Published:** 2021-04-28

**Authors:** Benjamin Seyd, Henri Band

**Affiliations:** 1 Friedrich-Schiller-Universität Jena, Jena, Deutschland; 2 Berlin, Deutschland

1992 war es, als der demokratische Kandidat Bill Clinton unter dem Slogan „It’s the economy, stupid!“ ganz auf die Karte Wirtschaftskompetenz setzte, um den Golfkriegshelden George W. Bush als US-Präsident abzulösen. Es waren zugleich die Gründungsjahre des Berliner Journals für Soziologie, das sich in besonderer Weise den Folgen und Begleiterscheinungen der marktwirtschaftlichen Transformation im ehemaligen Ostblock widmete. Hier wie dort bestand die Erwartung, dass mit der richtigen wirtschaftspolitischen Ausrichtung die Früchte der Marktwirtschaft auch jenen zugutekommen könnten, denen ein Leben in Wohlstand, Freiheit und Sicherheit bis dahin verwehrt geblieben war.

Bald dreißig Jahre später ist gesellschaftliche Transformation immer noch eines der Schlüsselthemen, denen sich unsere Zeitschrift verschrieben hat – doch die Vorzeichen haben sich geändert. Niedrige Wachstumsraten, eine gewisse Desillusionierung über die Globalisierung und ihre Folgen, die Große Pandemie und nicht zuletzt die Einsicht in das Ausmaß ökologischer Gefährdungen haben dazu beigetragen, dass dem Kapitalismus selbst eine Art von Transformation ins Haus stehen könnte. Gleichzeitig hat das uneingelöste Versprechen von Freihandel, Wachstum und „trickle-down“ dazu geführt, dass Verteilungsfragen wieder in anderer, expliziterer Form gestellt werden. Was in der Clinton’schen Losung bestenfalls mitschwang, ist spätestens mit der globalen Finanzkrise wieder ins Herz politischer und sozialwissenschaftlicher Auseinandersetzungen gerückt: It’s inequality, stupid!

In der Vervielfältigung, Vertiefung, Verfestigung und Verarbeitung gesellschaftlicher Ungleichheiten, wie sie zahlreiche Studien und Reports belegen, lassen sich die Konturen einer Wiederbelebung der sozialen Frage ausmachen. In der Soziologie findet das seinen Niederschlag in Instituten für gesellschaftlichen Zusammenhalt oder einem neugegründeten SFB zum Strukturwandel des Eigentums, in der Wiederentdeckung der Klassentheorie oder einer Flut an Publikationen, die den sozialen Ursachen politischer Polarisierungstendenzen nachgehen. Darüber hinaus ist Ungleichheit auch dort mit im Spiel, wo es vordergründig um andere Probleme geht: Ob Klimawandel, Ressourcenkonflikte, psychische Belastungen oder SARS-CoV‑2 – immer wären die Probleme eher zu bewältigen, wenn alle gleichermaßen von ihnen betroffen und an ihrer Lösung interessiert wären. Wenn es stimmt, dass Autonomie das „Hypergut“ der Moderne ist, könnte Ungleichheit ihr Hyperproblem sein.

Allerdings birgt so viel thematische Omnipräsenz ihre eigenen Tücken. Führt sie doch in Versuchung, die sichtbar werdenden Spaltungen vorschnell für evidenter und wirkmächtiger zu halten als sie womöglich sind und von allzu eindeutigen Grenzziehungen auszugehen: zwischen Gewinnern und Verlierern der Globalisierung, zwischen reichem Norden und armem Süden, zwischen weltoffenen urbanen Eliten und gemeinschaftsorientierten Landbewohnern. Mögen das im Einzelfall nützliche Zuspitzungen sein, laufen solche Polarisierungen gleichwohl Gefahr, unterkomplexen Schlüssen Vorschub zu leisten. Deshalb interessieren Soziolog_innen nicht nur die großen Linien des sozialen Wandels, sondern auch seine feinen Verästelungen. Zur zeitdiagnostischen Pointierung hat akribische Begriffsarbeit und genaue Empirie zu treten.

Vom „Interventionsrecht der Empirie“ Gebrauch zu machen, davon sprechen auch Steffen Mau, Thomas Lux und Fabian Gülzau.^1^ In ihrem Beitrag, der das vorliegende Heft eröffnet, gehen die drei Autoren der Frage nach, ob sich die in den öffentlichen Debatten und zeitdiagnostischen Texten oft unterstellte neue Lagerbildung zwischen den ökonomisch Bessergestellten, die sich zugleich für Migration und Diversität aussprächen, einerseits und den ökonomisch Schlechtergestellten, die gegen Migration und Diversität eingestellt wären, andererseits empirisch erhärten lässt. Die Autoren können zeigen, dass es sich bei den drei Konfliktthemen Ökonomie, Migration und Diversität um relativ eigenständige Dimensionen handelt, was sich in nicht konform laufenden ungleichheitskritischen bzw. -affirmativen Einstellungen innerhalb der Statusgruppen widerspiegelt.

Angesichts des sich augenscheinlich vergrößernden Abstands zwischen oben und unten ist die Frage von besonderem Interesse, wer „die da oben“ eigentlich sind, woher sie kommen und wie es sich mit ihrer Mobilität, Loyalität und Globalität verhält. Michael Hartmann geht diesen Fragen schon seit vielen Jahren nach. Wenn er in seinem Beitrag zu den Rekrutierungswegen der deutschen Wirtschaftselite feststellt, dass es an der Spitze wenig Neues gibt, ist das allerdings nicht halb so unspektakulär, wie es zunächst klingt. Hartmann findet nicht nur bestätigt, dass der Erfolg den Unternehmenschefs buchstäblich in die Wiege gelegt ist – immer noch stammen vier Fünftel der Vorstandsvorsitzenden aus Familien des Bürger- und Großbürgertums –, sondern dass sich diese Tendenz mit dem weiteren Relevanzverlust einer betrieblichen Lehre sogar zu verstärken droht. Allerdings sind, anders als in den Einlassungen zum „Ende der Deutschland AG“ vorhergesagt, Hauskarrieren noch immer typisch und Ingenieure im Chefsessel nach wie vor gern gesehen. Stattdessen sind die einst dominierenden Juristen weg vom Fenster deutscher Chefetagen – und zwar fast völlig! Wer hätte das gedacht?

Weit weg von den Chefetagen, gewissermaßen ganz am anderen Ende des Fahrstuhlschachts, finden sich allzu häufig Familien mit Migrationshintergrund. Das ist eine bereits vielfach belegte Tatsache; nicht so genau verstanden sind aber die Mechanismen der Armutstransmission von Generation zu Generation. Janina Zölch und Petra Böhnke gehen in ihrem Beitrag der Frage nach, wie die innerfamilialen Lebenslagen, Beziehungen und Orientierungsmuster die Weitergabe von Armut bedingen und unter welchen Konstellationen der Migrationshintergrund derlei Effekte zusätzlich verstärken kann. Die Autorinnen konstatieren für die von ihnen untersuchten Familien, dass sich die Benachteiligungserfahrungen aus der Armuts- und aus der Migrationssituation wechselseitig potenzieren und so die Entwicklung jener alternativen, proaktiven Handlungsmuster erschweren, die nötig sind, um der Armutssituation aus eigener Kraft zu entkommen.

Wo Armut ist, wird auch Gewalt wahrscheinlicher. Warum nicht nur Ungleichheit, sondern auch Gewalt nicht aus der Welt zu schaffen scheint, ist eine hochkontroverse Frage. Schon seit Längerem zieht sich durch die deutsche Gewaltsoziologie daher ein Streit zwischen den sogenannten „Innovateuren“ und „Mainstreamern“ um ein soziologisch angemessenes Gewaltverständnis. Zur Entspannung und Versachlichung der zum Teil verhärteten Debatte um den einzig richtigen Gewaltbegriff und um phänomenale, situationale und/oder strukturelle Gewalterklärungen empfehlen Thomas Kron und Lena M. Verneuer der Zunft ein soziologisches Wannenbad: Das bewährte struktur-individualistische Grundmodell („Badewannenmodell“) der soziologischen Erklärung erlaube es immer noch am besten, die in der Debatte oftmals einseitig hervorgehobenen Teilaspekte des zu erklärenden Phänomens zu integrieren und fallspezifisch zu modellieren. Universalerklärungen würden der Vielgestaltigkeit und Vielschichtigkeit von Gewaltphänomenen dagegen nicht gerecht.

Einer ganz anderen Frage widmet sich schließlich die letzte Abhandlung des Heftes aus der Feder von Léa Renard und Benedicte Zimmermann. Aus der Überzeugung heraus, dass Arbeit wenn schon nicht reich, so doch wenigstens nicht krank und möglichst sogar zufrieden machen sollte, ist „Gute Arbeit“ zu einer ebenso zentralen wie populären Forderung deutscher Gewerkschaften geworden. Dabei knüpfen Letztere an eine europäische Debatte an, die anderswo in vergleichbarer Weise Nachhall findet: In Frankreich werden ähnliche Fragen unter dem Stichwort der „qualité de vie au travail“ diskutiert. Doch wie die Autorinnen mit dem feinkalibrigen Instrumentarium der „histoire croisée“ nachzeichnen, zeigen sich auch bei den Überwindungsideen von Ungleichheit feine Unterschiede. Entscheidend sind dabei – wie so häufig in politischen Konflikten – abweichende Problemdefinitionen. Diese nachzuzeichnen, hilft nicht nur zu verstehen, worum sich der Streit um bessere Arbeitsverhältnisse beiderseits des Rheins dreht, sondern auch, worauf es ankommt, will man der fortgesetzten Polarisierung zwischen Vermögenden und Lohnabhängigen Einhalt gebieten.

Den Abschluss des Heftes bildet ein Nachruf von Hans-Peter Müller auf Guenther Roth, der sich u.a. durch seine profunden Beiträge zu Max Weber auch in Deutschland einen Namen gemacht hat. Den 100. Todestag *des* Klassikers der Soziologie haben wir keineswegs vergessen. Den Auftakt des 31. Jahrganges wird ein Schwerpunktheft zu Max Weber und der erfolgreichen Fertigstellung der Max Weber-Gesamtausgabe bilden. In den daran anschließenden Ausgaben wird sich unser Blick u.a. auf die Renaissance der Klassentheorie und den Zustand der Globalisierung richten. Und vorher noch, im Rahmen eines Schwerpunkts zum Goffman’schen Konzept des „cooling out“, auf den Umgang mit Scheitern: Angesichts der bisherigen Bilanz in Sachen Ungleichheit auch kein ganz fernliegendes Thema.

## Footnotes

1 Und zwar in einem Tweet zu ihrem Artikel auf Twitter, wo seit kurzem auch das Berliner Journal für Soziologie aktiv ist: twitter.com/BerlinerJournal.

